# Analysis of the Warpage Phenomenon of Micro-Sized Parts with Precision Injection Molding by Experiment, Numerical Simulation, and Grey Theory

**DOI:** 10.3390/polym14091845

**Published:** 2022-04-30

**Authors:** Wei-Chun Lin, Fang-Yu Fan, Chiung-Fang Huang, Yung-Kang Shen, Hao Wang

**Affiliations:** 1 School of Dental Technology, College of Oral Medicine, Taipei Medical University, Taipei 110, Taiwan; weichun1253@tmu.edu.tw (W.-C.L.); fish884027@tmu.edu.tw (F.-Y.F.); chiung0102@tmu.edu.tw (C.-F.H.); 2 Research Center for Biomedical Devices, Taipei Medical University, Taipei 110, Taiwan; 3 Division of Family and Operative Dentistry, Department of Dentistry, Taipei Medical University Hospital, Taipei 110, Taiwan; 4 Department of Mechanical Engineering, Faculty of Engineering, National University of Singapore, Singapore 119077, Singapore; mpewhao@nus.edu.sg

**Keywords:** warpage, precision injection molding, optimal design and processing, experiment, numerical simulation, grey theory

## Abstract

In this study, we determined the effects of design and processing parameters of precision injection molding (PIM) to minimize warpage phenomena of micro-sized parts using various plastics (polyoxymethylene (POM), acrylonitrile-butadiene-styrene (ABS), polypropylene (PP), polyamide (PA), and ABS+ polycarbonate (PC)). We applied a numerical simulation (Moldflow) to determine the runner’s balance in multi-cavities of the micro-sized part and simulate the warpage phenomenon of micro-parts with PIM. We used simulation data to fabricate a steel mold by computer numerical control (CNC) machining. In this, we study manufactured a micro-sized part and measured its warpage value using various PIM process parameters (melt temperature, mold temperature, injection pressure, and filling time). In order to obtain optimal results (i.e., minimum warpage), we employed the Taguchi method and grey theory to discern the influence of each process parameter on PIM. Finally, we determined that the most significant PIM process parameter influencing the warpage phenomenon of micro-sized parts was the mold temperature, regardless of whether in terms of the experimental results, numerical simulations, or grey theory. The PA material had the most suitable properties for application for micro-sized parts, regardless of whether in terms of experimental results, numerical simulations, or grey theory for PIM. This study also illustrates that micro-sized parts can be fabricated by PIM without the use of micro-injection molding, and we determined that the mold temperature required for molding does not need to be higher than the glass-transition temperature of the material.

## 1. Introduction

Precision injection molding (IM; PIM) is an important technology that can increase productivity and reduce costs in fields such as electronics, photonics, and medical plastics by allowing molded plastics to replace more expensive metal and ceramic components. Higher melt and mold temperatures, as well as higher injection speeds (shear), increase melt flow, which improves precision [1]. The size of micro-flow marks is related to the melt temperature, mold temperature, no-flow temperature, the thermal diffusivity of the resin, and time pitch formation of flow mark ridges and valleys in IM [2]. The surface waviness of lenses can be reduced with higher values of melt temperature, injection pressure, packing pressure, and mold temperature during IM. The most significant factor was determined to be the melt temperature, followed by the packing pressure, injection pressure, and mold temperature [3]. The maximum residual stress of micro-lens arrays decreased as the melt temperature, mold temperature, packing pressure, and cooling time increased. The maximum residual stress increased as the flow rate increased [4]. Variations in the refractive index of injection-molded polymethyl methacrylate (PMMA) optical lenses were more uniform when higher packing pressures were used but less so for lower packing pressures [5]. Multiple objective functions reflecting the product quality, manufacturing costs, and molding efficiency were utilized to construct an optimization model of IM parameters [6]. This requires high optical quality with high form accuracy and lower residual stresses, which are challenges for both optical tool insert machining and PIM processes [7]. IM process parameters have significant effects on the optical performance and surface waviness of precision plastic optical lenses. An orthogonal experiment was carried out with the Taguchi method, and the results were examined by an analysis of variance (ANOVA) [8]. The proposed control method resulted in a decrease in product weight variations from 0.16% to 0.02% in the case of varying the mold temperature, and the number of cycles to return stability decreased from 11 to 5 with respect to variations in the melt temperature [9]. An overview of various studies related to research on the topic of monitoring and control systems for IM explained why the application of artificial intelligence (AI) methods is beneficial [10]. Micro-sized polymer parts can usually be manufactured either by conventional IM or by micro-IM (µIM). Experimental validation of a functional analysis was carried out by molding the same micro-sized medical part of a thermoplastic elastomer material using the two processes by means of multi-cavity molds [11]. IM is a molding technology that melts material with the aid of a screw and an external heating device and then injects it into a mold to form the corresponding product as the mold cools [12]. Dimensional control and online defect detection are extremely important for quality control, particularly for high-resolution PIM [13]. A particle swarm optimization algorithm contributed to the tuning of hyperparameters of a support vector classifier (SVC) model in order to minimize the error between the SVC and experimental results in IM [14]. One report reviewed recent studies on methods for detecting relevant physical variables, optimizing process parameters, and determining control strategies of machine variables in the molding process [15]. The methodology presented the potential of reducing or eliminating the defect rate caused by material variations while allowing dimension predictions for injection-molded parts [16]. PIM of high-performance components requires that primary error sources that affect the molded component be identified and isolated so that these errors can be reduced if needed [17]. An appropriate choice of process parameters is essential in ensuring the precision and uniformity of molded parts [18]. To minimize part warpage, a segmented dynamic mold temperature control was developed with the aim of homogenizing the specific volume of a plate-shaped geometry [19].

µIM is applied to manufacture micro-sized parts and is among the most common and versatile methods mass production of complex plastic parts. The replication properties of products are discussed in terms of different µIM process parameters, which are mainly influenced by the mold temperature [20,21,22,23,24,25], holding pressure [21,24,25,26], injection velocity [23,24,27], and melt temperature [27].

There has been much research investigating warpage in IM by experimentation, numerical simulations, and soft computing. Polycarbonate (PC)/acrylonitrile–butadiene–styrene (ABS) composites exhibited 14.02% reduced warpage after optimization of processing parameters by numerical simulation using the Taguchi method [28]. The multi-response optimization of IM with polypropylene (PP) emphasized multiple response considerations using warpage [29]. A part’s warpage can be analyzed using various µIM process parameters (melt temperature, packing pressure, packing time, and cooling time). It was found that warpage properties are mainly influenced by the cooling time [30,31,32], melt temperature [31,32,33,34,35,36,37], and packing pressure [31,33,34,36,38]. The packing time, cooling time, and melt temperature were the most significant factors influencing warpage reductions based on design of experiment (DOE), response surface methodology (RSM), firefly algorithm (FA), and annealing treatment [32].

The Michelson interferometric system is an effective approach to measure micro-sized parts manufactured by PIM. The application and theory of Michelson interferometry are well described in the literature [39,40,41,42,43,44,45,46,47].

Grey theory is an algorithm used to find optimal solutions in various situations. Deng [48] developed the theory for systems that lack information, such as structured messages, operating mechanisms, and behavior documents, which are referred to as grey systems. A grey relational analysis using expert systematic manipulations and subsequent data can resolve the effects of numerous variables [49]. An orthogonal array with a grey relational analysis and fuzzy logic was employed to determine a part’s warpage with various injection mold parameters (mold temperature, melt temperature, filling pressure, and filling time) [50,51]. The warpage property was found to mainly be significantly influenced by the mold temperature [52,53], melt temperature [54], and packing pressure [54,55].

In this study, we fabricated micro-sized parts using various plastic materials (polyoxymethylene (POM), ABS, PP, polyamide (PA), and ABS+ PC) by PIM. The PIM process parameters we examined were the melt temperature, mold temperature, injection pressure, and filling time. We simultaneously compared the warpage phenomenon of PIM-produced micro-sized parts with experimentation, numerical simulations, and grey theory. In this study, we also discuss the mold window of micro-sized parts for various materials in PIM. The warpage of molded micro-sized parts was measured on a charge-coupled device (CCD) and laser equipment. The aim of this study was to apply the Taguchi method to determine the minimum warpage of PIM-produced micro-sized parts, and we mainly attempted to use PIM instead of µIM to produce precision-molded parts.

## 2. Materials and Methods

### 2.1. Experimental

#### 2.1.1. Precision Injection Molding (PIM)

**Figure 1:**
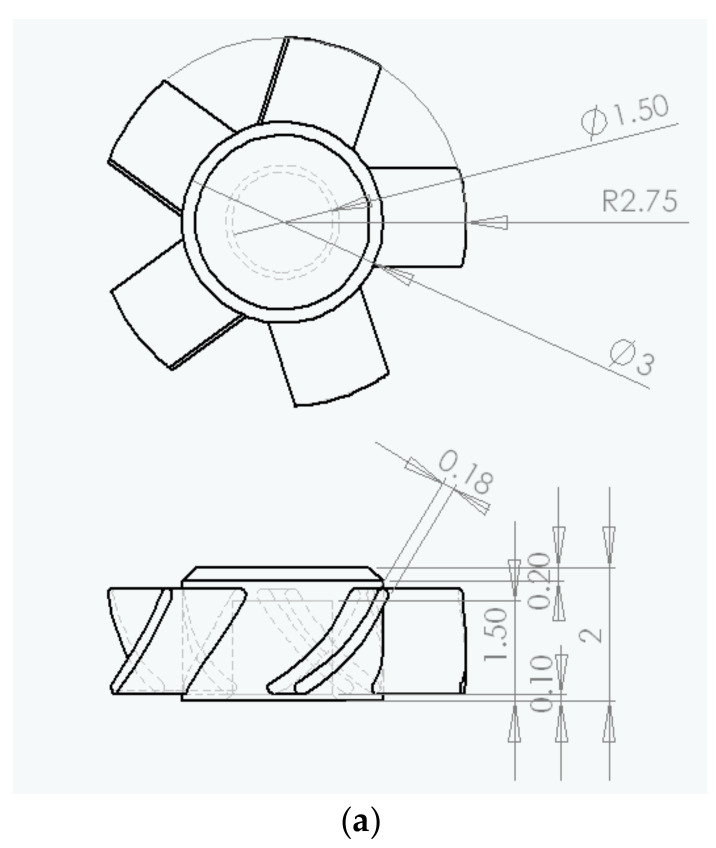

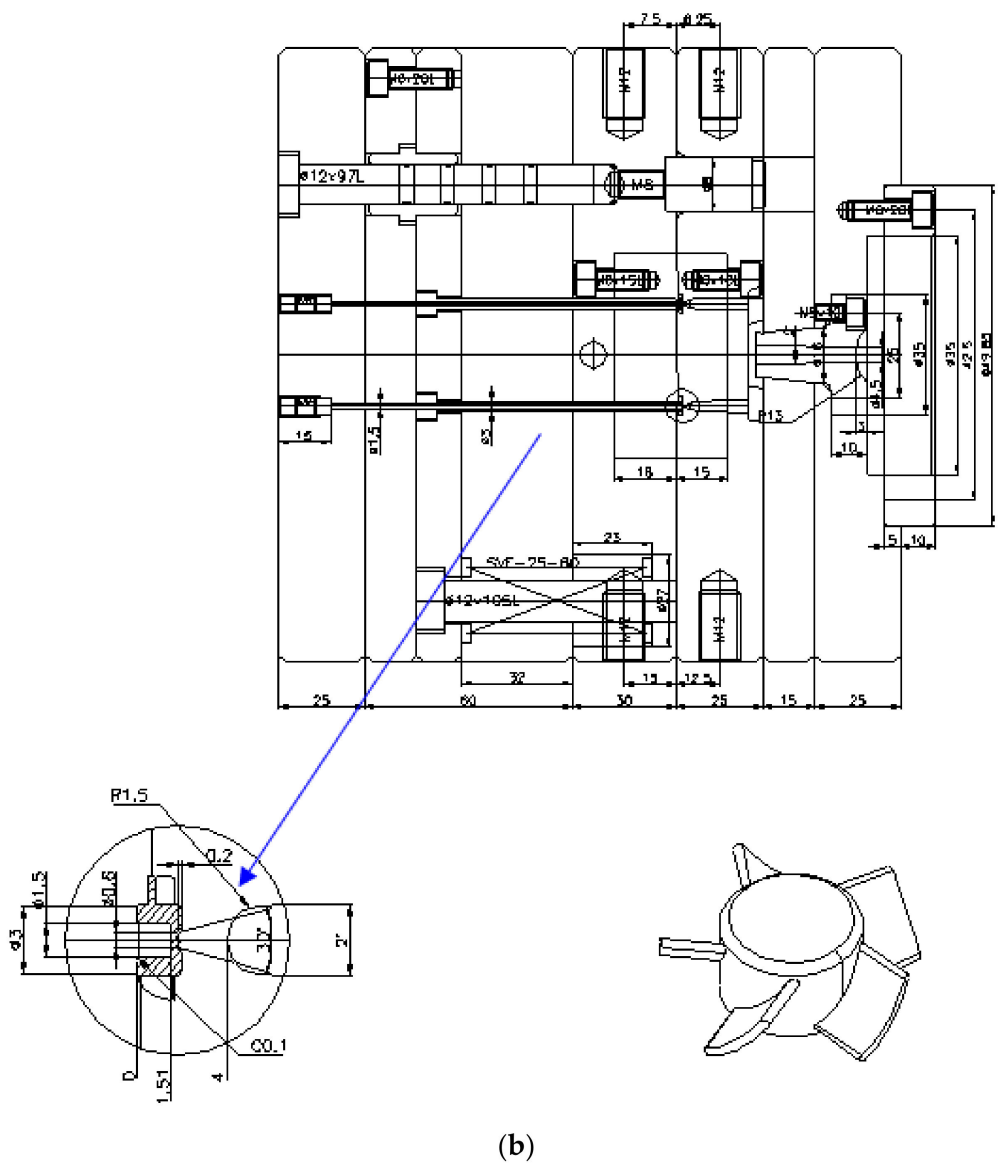
The dimensions of the micro-sized part and mold for PIM (unit: mm). (**a**) Micro-sized part (micro-electric fan) and (**b**) mold (side view).

Figure 1 indicates the dimensions of the micro-sized part (a micro-sized electric fan as the micro-sized case) and its mold. The thickness of the blade of the micro-sized part (micro-sized electric fan) was 180 μm. We employed PIM to fabricate the micro-sized part and attempted to use PIM without the use of µIM techniques.

**Figure 2:**
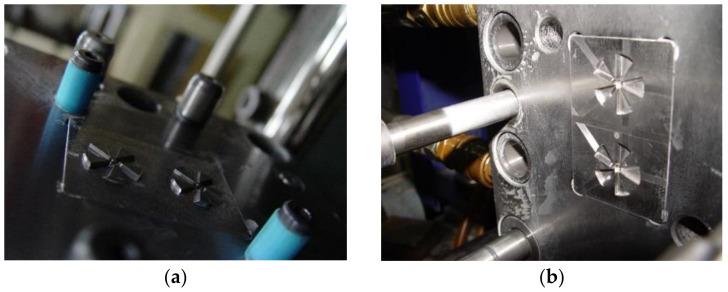
Cavity of the mold insert. (**a**) Cavity for moving part. (**b**) Cavity for stationary part.

A three-plate mold with two cavities was utilized during PIM. The mold cavities were fabricated by a computer numerical control (CNC) process. The mold material we used was NAK-80, and its hardness was 350 HMV. The inlet gate of the three-plate mold was a sidewall pin gate. The mold cavities of moving and stationary parts are shown in Figure 2. A PIM machine (220S, Arburg, Lossburg, Germany) with a screw diameter of 18 mm and a clamping force of 25 tons was used for all experiments. A Regloplas 300S (Switzerland) mold temperature control machine with a precision of ±1 °C was used. We used POM (Delrin 9255, DuPont Engineering Polymers, Midland, MI, USA), ABS (SP-6, Chi Mei, Taiwan), PP (Polypro BC1, Mitsubishi Chemicals, Japan), PA (X7323 Trans, Creanova-Trogqmid, Memphis, TN, USA), and ABS + PC (Novodur KU 2-5300, Bayer USA, Boston, MA, USA) materials to fabricate the micro-sized parts via PIM. In this study, we investigated warpage phenomena of PIM-fabricated micro-sized parts in terms of optimal processing parameters and optimal materials. The PIM process parameters were the melt temperature (A), mold temperature (B), injection pressure (C), and filling time (D). Table 1 lists values of the PIM process parameters for the various plastic materials. To identify the relative significance of these four process parameters, various experiments were performed with 3^4^ runs. A statistics-based experimental design method, the Taguchi method [56], was utilized to reduce the number of experimental runs (Table 2). In this study, we also investigated the mold window of micro-sized parts using various materials in PIM to find a suitable material.

#### 2.1.2. Measurement (Warpage)

**Figure 3:**
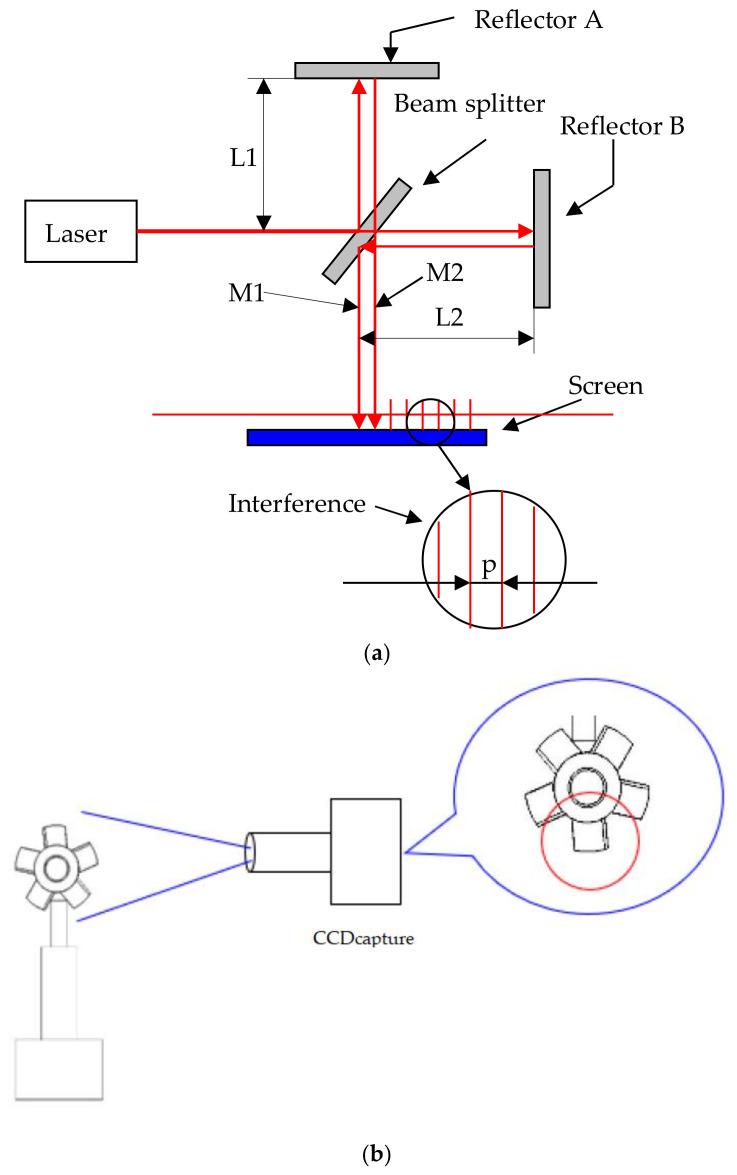

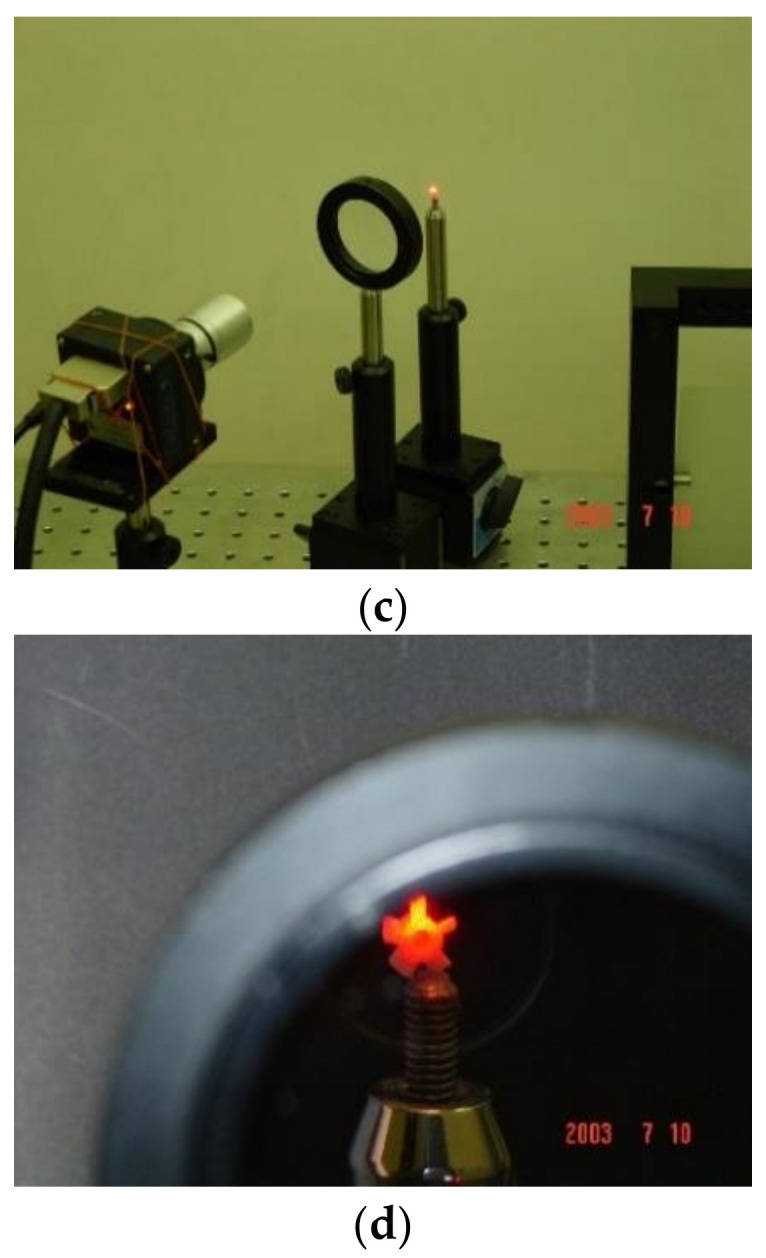
Schematic diagram of Michelson interference and measurement equipment assembly platform. (**a**) Arrangement of measurement equipment. (**b**) Micro-sized part and CCD system. (**c**) Real measurement system and micro-sized part. (**d**) Laser light on the surface of the micro-sized part.

The allowance error of a fabricated micro-sized part is very important. Analysis of warpage phenomena of micro-sized parts was the primary task of this study. We used optimal processing parameters to determine the relationship between PIM process parameters and warpage of the micro-sized parts. Laser equipment (red-light He-Ne laser, 25 mW) and a charge-coupled device (CCD; A102K, Basler, Sony, Tokyo, Japan) were employed to measure the warpage of the micro-sized parts (Figure 3). A three-dimensional (3D) laser scanner (LSH-II-150, Hawk, Nextc, Corvallis, OR, USA) was applied to determine the extent of warpage of the micro-sized parts.

In the measurement procedure, due to the small geometric size of the actual micro-sized parts, the warpage of the could not be measured with a conventional method. Therefore, the measurement method of optical interference fringe projection was used to determine whether the actual micro-sized parts were warped. We used the Michelson interference fringe. The laser beam was divided into two lights by a beam splitter and a mirror to produce bright and dark interference fringes, as shown in Figure 2, due to the overlap of the two reflected light beams. L1 is the distance from mirror A to the beam splitter, L2 is the distance between mirror B and the beam splitter, M1 is reflected light from mirror A, M2 is reflected light from mirror B, and p is the interference fringe pitch. When the laser light hits the beam splitter and is separated in two, it hits reflector A to generate reflected light M1 and hits reflector B to generate reflected light M2, which are projected onto a screen to generate interference fringes. Reflector B was fine-tuned to reflect lights M1 and M2 so that there was an angle between them. With a smaller distance, the stripe pitch, p, is larger. When the distance, L1, from the mirror to the beam splitter is not equal to L2, the contrasting effect worsens. When L1 is equal to L2, the contrast is optimal. When the interference fringes hit the micro-sized part, the stripes projected onto the micro-sized part bend because of fluctuations in the micro-sized part’s surface. This phenomenon was used to determine whether the micro-sized part was warped. During the imaging process, the color of the finished micro-sized part (a millimeter-size fan) affected the imaging, as a deeper color of the micro-sized part (micro-electric fan) produced a better imaging effect. Because the micro-sized part made of PA was transparent after PIM, it was necessary to use a singular pen to darken the imaged part to facilitate imaging. We demonstrated that the Michelson interferometer design for position measurements was capable of a fringe interpolation accuracy of 1 part in 36,000.

**Figure 4:**
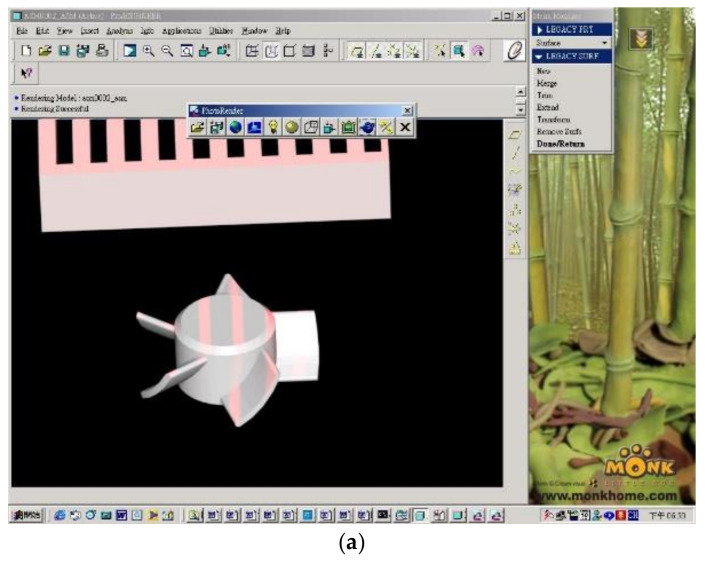

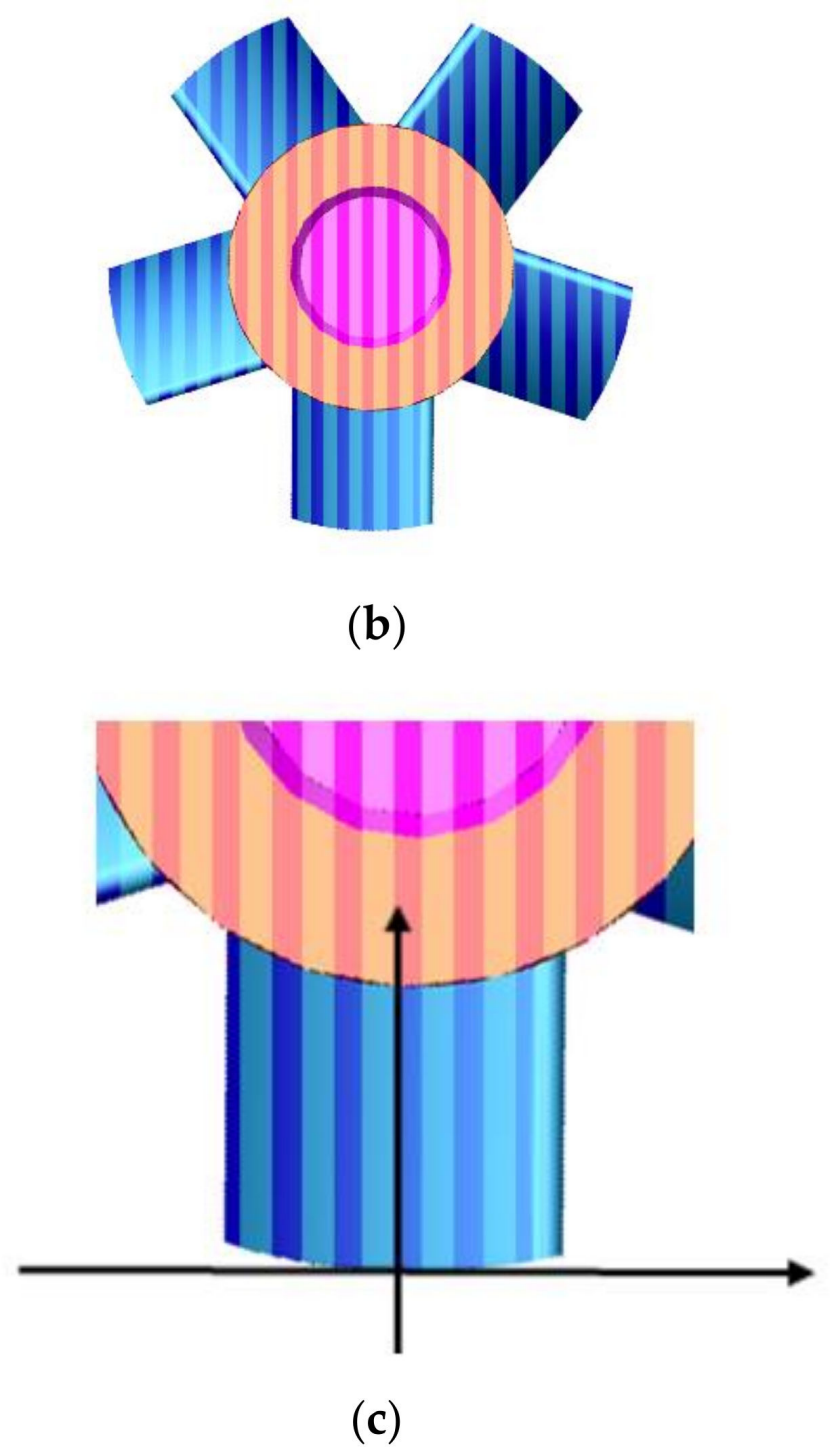
Textures on the surface of micro-sized part by laser scanning. (**a**) Laser light scans the surface of the micro-sized part and transfers the image to the software system. (**b**) The theoretical texture of the surface of the micro-sized part. (**c**) The theoretical texture of the surface of the micro-sized part (enlarged).

Figure 4 shows the theoretical texture of the micro-sized part’s surface. One of the fan blades was fixed vertically on a pole using quick-drying glue. After CCD imaging, an enlarged and inverted image was formed. The circled part was caused by interference fringes of the projection site. As shown in the Figure 4, on the blade part, because the blade radian was formed by rotation and extension in the x direction as the axis, the interference fringe was parallel to the x direction and was straighter than the y direction. In theory, this fringe projection of the part should be a straight line at this location.

### 2.2. Numerical Simulation

Continuity equation:

(1) $$\frac{D\rho }{Dt}=\frac{\partial \rho }{\partial t}+\nabla \cdot \left(\rho \vec{u}\right)=0$$

Momentum equation:

(2) $$\rho \frac{D\vec{u}}{Dt}=-\nabla p+\eta \nabla^{2}\vec{u}+\rho \vec{g}$$

Energy equation:

(3) $$\rho c_{p}\frac{DT}{Dt}=k\left(\nabla^{2}T\right)+\eta {\dot{\gamma }}^{2}$$

Then

$$\dot{\gamma }=\nabla \vec{u}\cdot \nabla \vec{u}$$

where *t* is time, *ρ* is density, $\vec{u}$ is the velocity vector, *P* is pressure, $\vec{g}$ is the gravity vector, *η* is viscosity, *c_p_* is specific heat, *k* is thermal conductivity, *T* is temperature, and $\dot{\gamma }$ is the shear rate.

The viscosity model of a fluid is described by the following equations:

(4) $$\eta \left(\dot{\gamma },T,P\right)=\frac{\eta_{0}\left(T,P\right)}{1+{\left(\frac{\eta_{0}\dot{\gamma }}{\tau^{*}}\right)}^{1-m}},$$

(5) $$\eta_{0}\left(T,P\right)=D_{1}\exp\left[-\frac{A_{1}\left(T-T^{*}\right)}{A_{2}+\left(T-T^{*}\right)}\right]\;,T<T^{*},$$

(6) $$\eta_{0}\left(T,P\right)=\infty ,T>T^{*}$$

(7) $$T*\left(P\right)=D_{2}+D_{3}P$$

and

(8) $$A_{2}={\tilde{A}}_{2}+D_{3}P$$

where *T*^∗^ denotes the glass-transition temperature of the polymer and *m* represents the flow index. *A*_1_, *A*_2_, *D*_1_, *D*_2_, and *D*_3_ are coefficients.

Boundary and initial conditions are described by the following equations:

(9) $$\vec{u}=0;\;T=T_{w};\;\frac{\partial P}{\partial n}=0\;\mathrm{at}z=\pm h\;(\mathrm{at the mold wall}),$$

(10) $$\frac{\partial \vec{u}}{\partial z}=\frac{\partial T}{\partial z}=0\;\mathrm{at}z=0\;(\mathrm{at the centerline}),$$

(11) P=0 (at the flow front), and

(12) $$\vec{u}=u(x,y,z,t)\;(\mathrm{at the inlet});$$

where *T_w_* is the mold temperature, *n* denotes normal direction, and *u* represents the inlet velocity.

The energy balance on a solid–liquid interface is described by:

(13) $$T_{s}=T_{l}=T_{m}\;at\;z=s\left(x,y,t\right)\;for\;t>0\;\mathrm{and}$$

(14) $$k_{s}\left(\frac{\partial T_{s}}{\partial n}\right)\left|{}_{z=s\left(x,y,t\right)}-\right.k_{l}\left(\frac{\partial T_{l}}{\partial n}\right)\left|{}_{z=s\left(x,y,t\right)}=\rho_{l}\right.L_{h}\frac{\partial s}{\partial t}$$

where *T_s_* is the solid temperature, *T_l_* is the liquid temperature, *T_m_* is the freezing temperature, *s* is the z-coordinate for the solid–liquid interface, *L_h_* is the latent heat, *k_s_* is the solid thermal conductivity, and *k_l_* is the liquid thermal conductivity.

**Figure 5:**
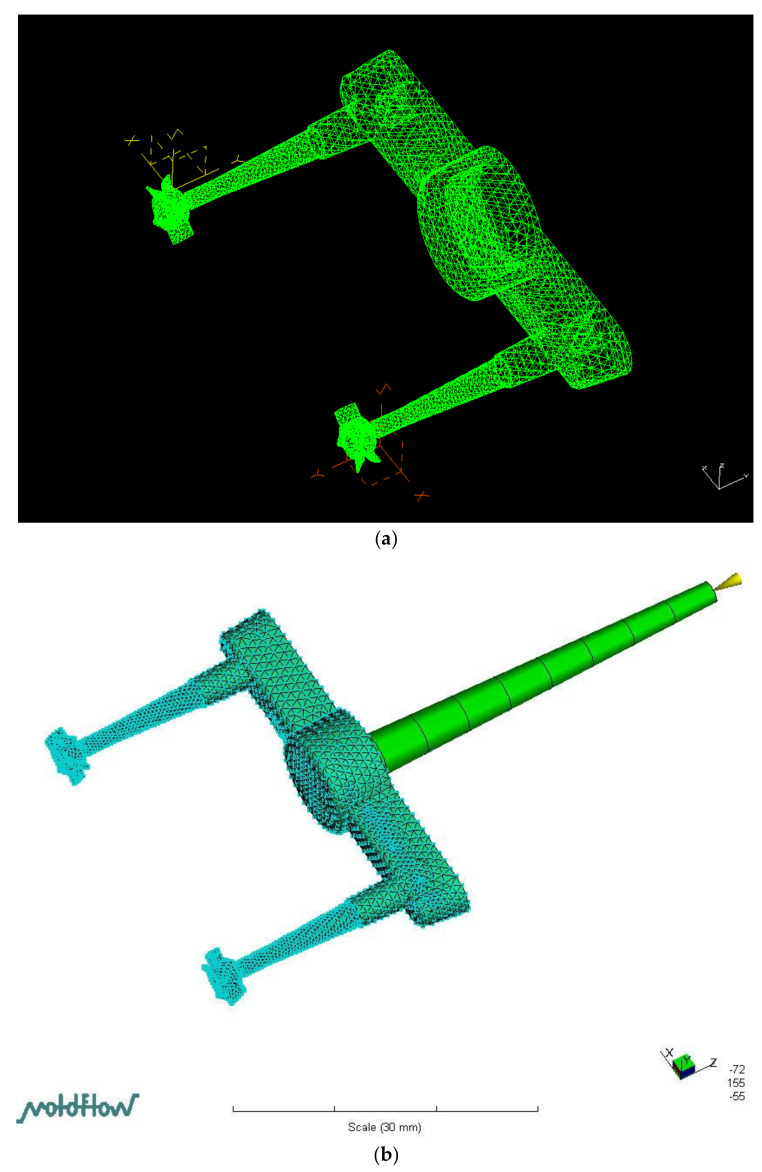
Meshing model by CAD (I-DEAS) and CAE (Moldflow). (**a**) Micro-sized part, sprue, runner, and gate system by CAD software drafting (I-DEAS). (**b**) Meshes of micro-sized part, sprue, runner, and gate system on Moldflow software.

The model contains (a) five independent variables of three velocities (*u*, *v*, and *w*), one pressure variable (*P*), and one temperature (*T*); and (b) one dependent variable, i.e., viscosity (η). The governing equations were solved using the control volume finite element method. For details of the numerical simulation, see Shen et al. [57]. We first employed computer-aided design (CAD) software (I-DEAS, vers. 12.0) to plot the full model (sprue, runner, gate, and micro-sized part) for PIM (Figure 5a). Computer-aided engineering (CAE) software (Moldflow, vers. 2015) was utilized to examine the PIM-fabricated micro-sized part (micro-electric fan) (Figure 5b). The 3D mesh used a four-node tetrahedral element for the numerical simulation. The simulation model had 54,646 meshes and 15,512 nodes on the micro-part. The calculation time was about 20 min for each case. A personal computer with a Pentium 6 3.5 GB central processing unit (CPU), 8 GB of memory, and a 1 TB hard disk was used.

### 2.3. Grey Theory

Grey theory [48] is mainly used to analyze relationships, establish models, and make decisions when a system model is not clear, the information is incomplete or unknown, or the operation status is unclear. In this study, we used a grey relational analysis based on grey theory to calculate the optimal PIM process parameters and applied grey decision analysis in grey theory to calculate the optimal material for the PIM-fabricated micro-sized part.

#### 2.3.1. Grey Relational Analysis of Optimal Processing Parameters

(1) Grey relational space.

Comparison sequence:

xi(k) = (xi(1),…, xi(k)) X(15)

and

Reference sequence:

xi(0)(k) = (xi(0)(1), …, xi(0)(k)) X(16)

where i = 0, …, m; k = 1, …, n N.

(2) The effect of the measurement method.

(a) Maximum: measurement of the upper-limit effect
  (17) $$x_{i}^{*}\left(k\right)=\frac{x_{i}^{\left(0\right)}\left(k\right)}{\max x_{i}^{\left(0\right)}\left(k\right)}$$
(b) Minimum: measurement of the lower-limit effect
  (18) $$x_{i}^{*}\left(k\right)=\frac{\min x_{i}^{\left(0\right)}\left(k\right)}{x_{i}^{\left(0\right)}\left(k\right)}$$
(c) Median: measurement of moderate effects-measurement of central effects
  (19) $$x_{i}^{*}\left(k\right)=\frac{\min\left\{x_{i}^{\left(0\right)}\left(k\right),x_{0}^{}\left(k\right)\right\}}{\max\left\{x_{i}^{\left(0\right)}\left(k\right),x_{0}\left(k\right)\right\}}$$

(3) Grey correlation measurement.
  (20) $$\gamma \left(x_{0},x_{i}\right)=\frac{1}{n}\sum_{k=1}^{n}\gamma \left(x_{0}\left(k\right),x_{i}\left(k\right)\right)$$
  where $\gamma \left(x_{0}\left(k\right),x_{i}\left(k\right)\right)$ and $\gamma \left(x_{0},x_{i}\right)$ are the grey correlation coefficient and degree, respectively.

An m × n matrix, X, was generated based on group sequences, $x_{i}^{*}{\left(k\right)}_{m}^{*}\left(m\right)$, by grey correlation.

(21) $$X=\left[\begin{array}{cccc} x_{1}^{*}\left(1\right) & x_{1}^{*}\left(2\right) & \cdots & x_{1}^{*}\left(n\right)\\ x_{2}^{*}\left(1\right) & x_{2}^{*}\left(2\right) & \cdots & x_{2}^{*}\left(n\right)\\ \vdots & \vdots & \ddots & \vdots \\ x_{m}^{*}\left(1\right) & x_{m}^{*}\left(2\right) & \cdots & x_{m}^{*}\left(n\right) \end{array}\right]$$

and

(22) $$\Delta =\left[\begin{array}{cccc} \Delta_{01}\left(1\right) & \Delta_{01}\left(2\right) & \cdots & \Delta_{01}\left(n\right)\\ \Delta_{02}\left(1\right) & \Delta_{02}\left(2\right) & \cdots & \Delta_{02}\left(n\right)\\ \vdots & \vdots & \ddots & \vdots \\ \Delta_{0m}\left(1\right) & \Delta_{0m}\left(2\right) & \cdots & \Delta_{0m}\left(n\right) \end{array}\right]$$

(23) $$\Delta_{0i}\left(k\right)=\left|x_{i}\left(k\right)-x_{0}\left(k\right)\right|$$

*i* = 1,…,m, with the smallest being Δmin and the largest Δmax.

Definition of the grey correlation coefficient $\gamma_{i}$:

(a) Locality when only $x_{0}(k)$ is the reference sequence and the other is the comparison sequence.
  (24) $$\gamma \left(x_{0}\left(k\right),x_{i}\left(k\right)\right)=\frac{\Delta_{\min}+\zeta \Delta_{\max}}{\Delta_{0i}+\zeta \Delta_{\max}}$$
  where $x_{0}\left(k\right)$ and $x_{i}\left(k\right)$ are reference sequence and a specific comparison sequence, respectively. *ξ* is the identification coefficient, and its value is between 0 and 1.
(b) Integrity: when any $x_{0}(k)$ is a reference sequence.
  (25) $$\gamma \left(x_{i}\left(k\right),x_{j}\left(k\right)\right)=\frac{\Delta_{\min}+\zeta \Delta_{\max}}{\Delta_{ij}\left(k\right)+\zeta \Delta_{\max}}$$
  (26) $$\Delta_{ij}\left(k\right)=∥x_{i}\left(k\right)-x_{j}\left(k\right)∥$$
  is the absolute value.

The grey correlation coefficient, *ξ*, is used to compare the background value with the measured micro-sized part and is generally 0.5.

(4) Grey correlation.

The quantitative measurement formula is called the grey correlation. The coefficient average value is taken to be:

(27) $$\gamma \left(x_{i},x_{j}\right)=\frac{1}{n}\sum_{k=1}^{n}\gamma \left(x_{i}\left(k\right),x_{j}\left(k\right)\right)$$

In an actual system, the grey relation of the extended formula is:

(28) $$\gamma \left(x_{i},x_{j}\right)=\sum_{k=1}^{n}\beta_{k}\gamma \left(x_{i}\left(k\right),x_{j}\left(k\right)\right)$$

where $\beta_{k}$ represents the normalized weight of factor *k*. When $\sum_{k=1}^{n}\beta_{k}=1$, the two equations are equal.

Finally, we determined the optimal PIM processing parameters.

#### 2.3.2. Grey Decision Analysis of Suitable Material

The grey decision was as follows: event **a** occurs, and countermeasure **b** is used to deal with it. This is the “situation”. Among multiple decisions dealing with the same event, the one with the best effect is chosen to deal with the event.

(1) Situation $S_{ij}=(a_{i}=b_{j})$ where $a_{i}$ is an event, and $b_{i}$ is a countermeasure.
(2) The target is an index for evaluating the situation effect (countermeasure effect), and an evaluation scale by an analytical hierarchy (AHP) table is shown in Table 3.
(3) Effect measurement for evaluating targets can be divided into three types.

(a) Measurement of the benefit target (upper-limit effect measurement)
  (29) $$r_{ij}=\frac{u_{ij}}{\max\left\{u_{ij}\right\}}$$
  where $r_{ij}$ is the situation, and $u_{ij}$ is the actual effect.
(b) Measurement of the cost target (lower-limit effect measurement)
  (30) $$r_{ij}=\frac{\min\left\{u_{ij}\right\}}{u_{ij}}$$
(c) Measurement of a specific target (specific-center effect measurement)
  (31) $$r_{ij}=\frac{\min\left\{u_{ij},u_{0}\right\}}{\max\left\{u_{ij},u_{0}\right\}}$$
  where $u_{0}$ is the actual effect.

(4) For situation $r_{ij}$, under the corresponding *M*, $M\left(u_{ij}^{p}\right)=r_{ij}^{p}$, if there is one target (p = 1, 2, …, l), where $r_{ij}^{1},r_{ij}^{2},\ldots ,r_{ij}^{l}$, $r_{ij}^{p}$ is called the comprehensive effect measurement, it is recorded as $r_{ij}^{\sum }$.

(32) $$r_{ij}^{\sum }=\frac{1}{l}\sum_{p=1}^{l}r_{ij}^{p}$$

Considering m countermeasures, $b_{1},b_{2},\ldots ,b_{m}$ are used to cope with event $a_{i}$, and there must be a corresponding comprehensive effect measurement vector, $r_{i}^{\sum }$:


(33) $$r_{i}^{\sum }=\left[r_{i1}^{\sum },r_{i2}^{\sum },.....,r_{im}^{\sum }\right]$$

Considering $i\in I=\left\{1,2,\ldots ,n\right\}$, there are *n* events, $i\in I=\left\{1,2,\ldots ,n\right\}$.

$r^{\sum }$ is the comprehensive effect measurement matrix.


(34) $$r^{\sum }=\left[\begin{array}{cccc} r_{11}^{\sum } & r_{12}^{\sum } & \cdots & r_{1m}^{\sum }\\ r_{21}^{\sum } & r_{22}^{\sum } & \cdots & r_{2m}^{\sum }\\ \vdots & \vdots & \ddots & \vdots \\ r_{n1}^{\sum } & r_{n2}^{\sum } & \cdots & r_{nm}^{\sum } \end{array}\right]$$

(5) Let $r_{i}^{\sum }=\left[r_{i1}^{\sum },r_{i2}^{\sum },\ldots ,r_{im}^{\sum }\right]$ be the comprehensive effect measurement vector of event $a_{i}$. If there are conditions, $r_{ij*}^{\sum }$, that meet $r_{ij*}^{\sum }=\max_{j}\;r_{ij}^{\sum }$, it is called a satisfactory situation $S_{{ij}^{*}}\left(a_{i}\cdot b_{j^{*}}\right)$. *n* is also called a satisfactory strategy, $b_{j^{*}}$, to deal with event $a_{i}$. A satisfactory $r_{i}^{\sum }$ is the optimal situation.

Using an L_9_ orthogonal table of the experiment to perform the grey correlation calculation and analysis, the grey correlation was degree discharged as a selection of the measurement sample, and it was compared to the experimental results.

The steps of grey decision analysis used in this study were as follows:

1. Values of five materials in the evaluation criteria were sampled.
2. Events, countermeasures, situations, goals, and samples were identified.
  Event: The material of the micro-sized part for PIM was selected as an event.
  Countermeasure: Schemes A, B, C, D, and E represent the POM, ABS, PP, PA, and ABS + PC materials, respectively.
  Therefore, plan A (b1), B (b2), …, E (b5).
  Situation:
  S11 = (a1, b1), material selection (scheme A)
  S12 = (a1, b2), material selection (scheme B)
  S13 = (a1, b3), material selection (scheme C)
  S14 = (a1, b4), material selection (scheme D)
  S15 = (a1, b5), material selection (scheme E)
3. Make effect measurements of effect samples.
4. Decide.
  Finally, we determined which material was the best mold material.

## 3. Results and Discussion

In order to obtain the best results for the process parameters and material selection for the micro-sized part with PIM (judged by the minimum warpage), the micro-sized part (micro-electric fan) had to be assembled on the central axis of the heat dissipation equipment, and the qualified warpage should be <0.5 mm.

### 3.1. Numerical Simulation (Also Compared to the Experiment on the Short Shot Situation)

**Figure 6:**
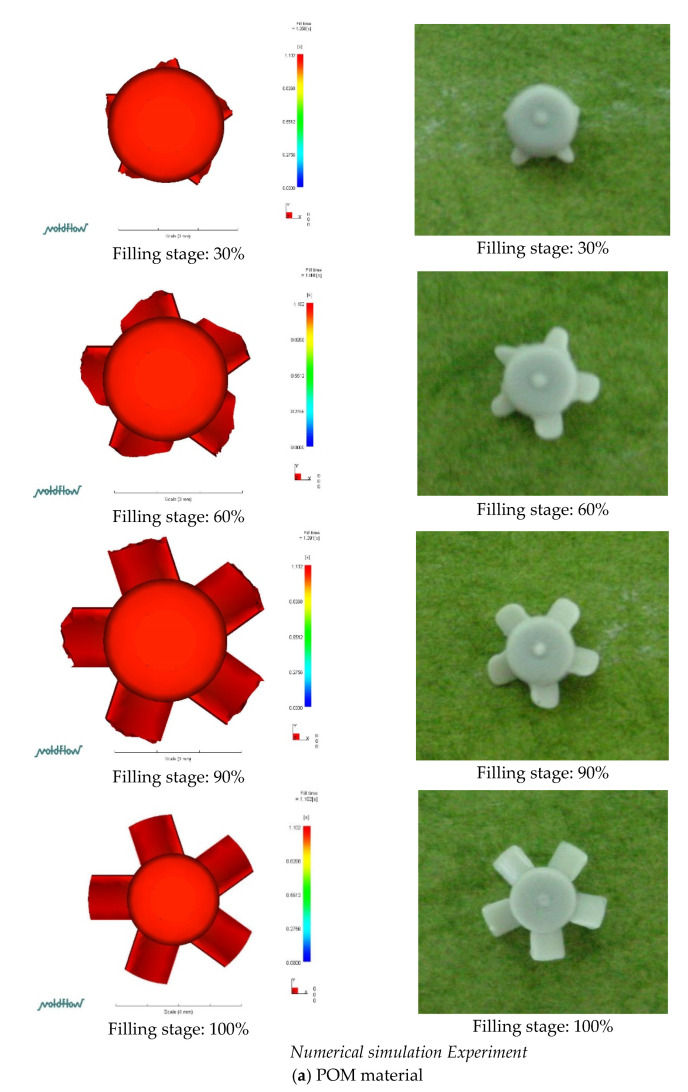

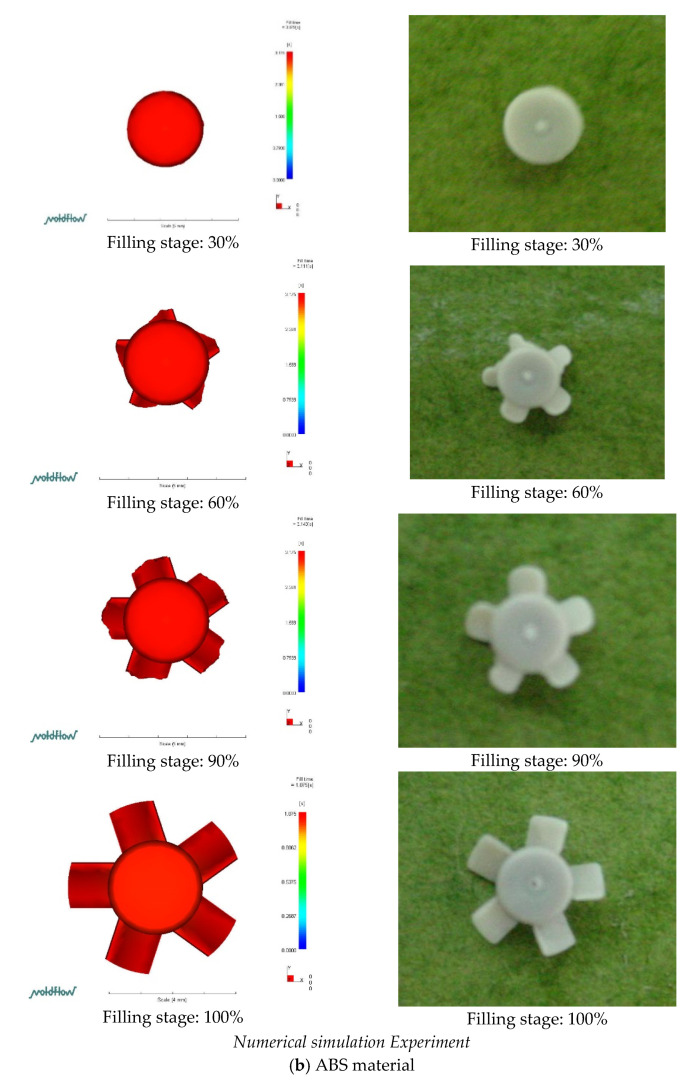

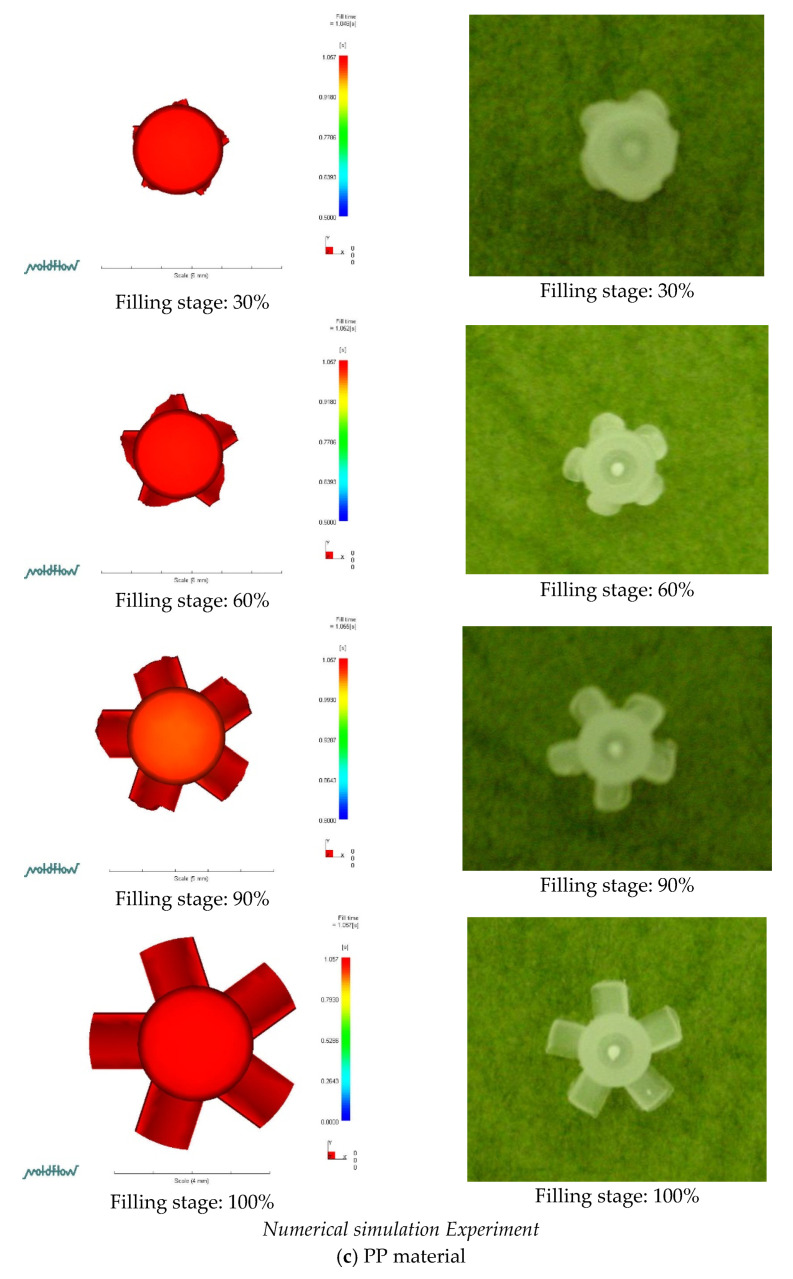

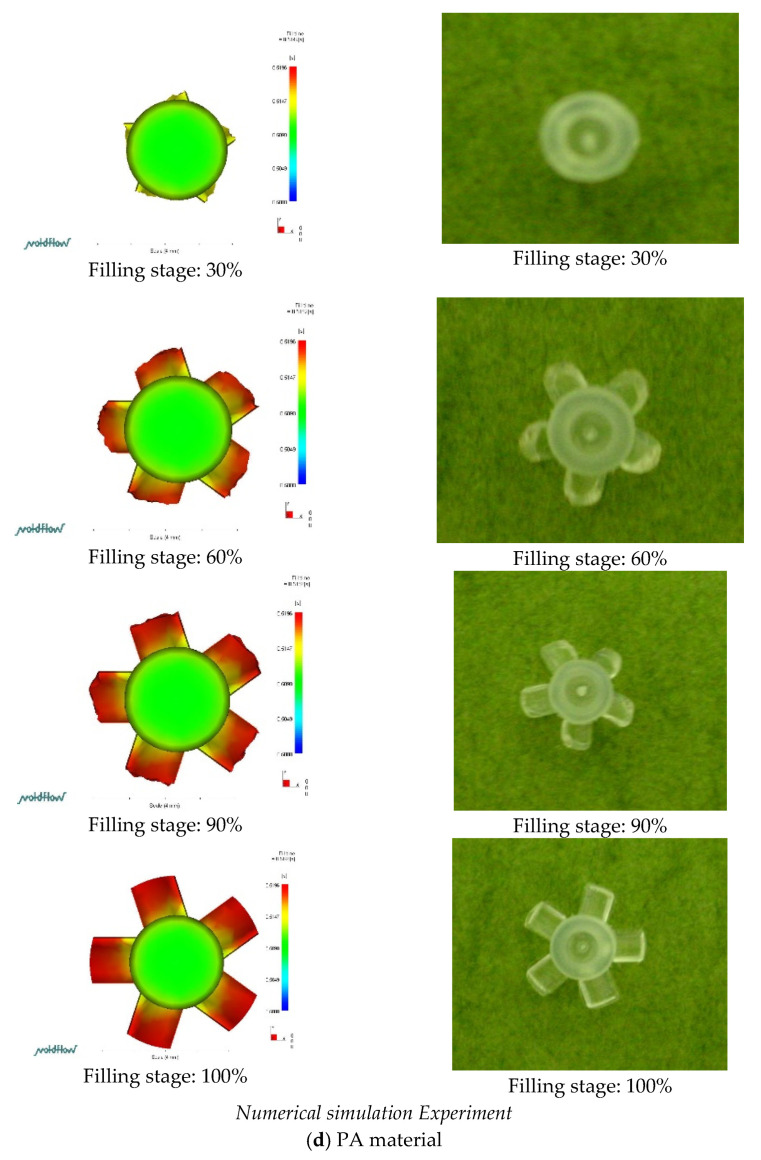

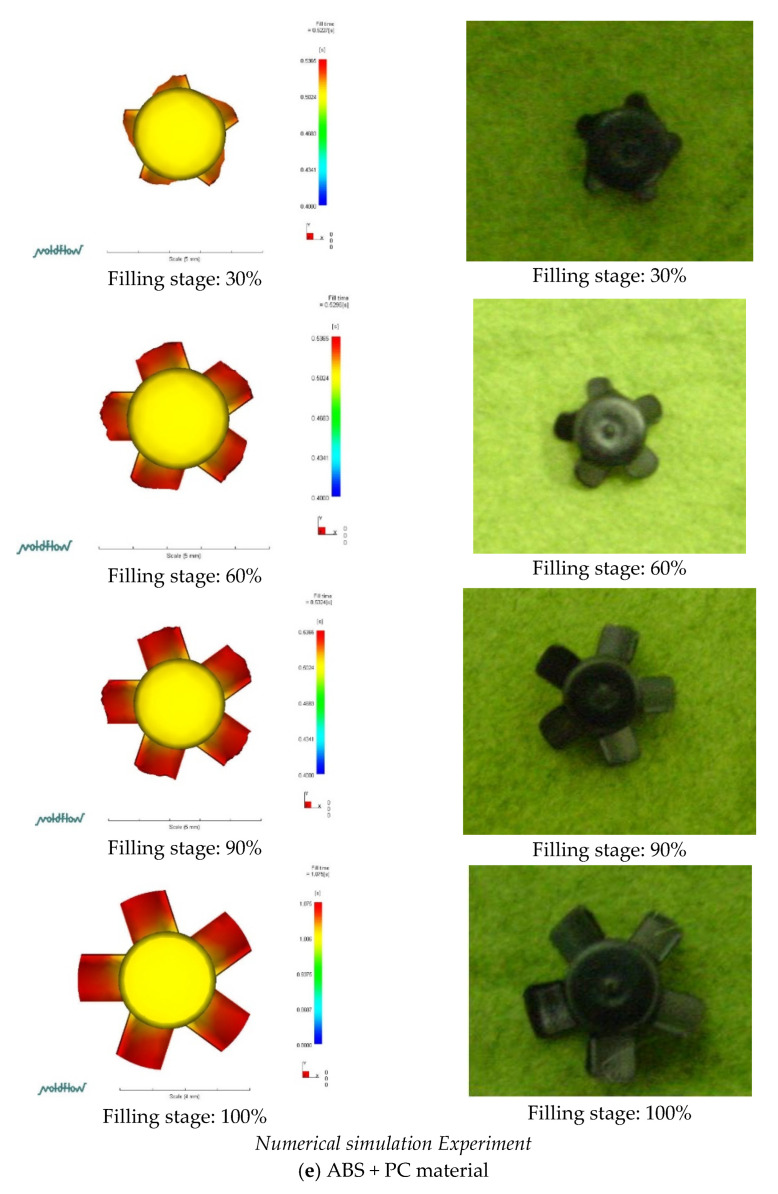
Short shot of micro-sized part with POM/ABS/PP/PA/ABS + PC material on PIM. (**a**) POM material; (**b**) ABS material; (**c**) PP material; (**d**) PA material; (**e**) ABS + PC material.

Figure 6 shows a short shot of the filling stage of PIM between the numerical simulation and experimental results for the micro-sized part (using POM/ABS/PP/PA/ABS + PC materials). Figure 6a indicates the simulation and experimental results of the PIM-fabricated micro-sized part using POM. The simulation and experimental results of the micro-sized part were similar in appearance, but the experimental results showed an asymmetric filling phenomenon at a 30% filling time. It was found that some blades of the experimental micro-sized part generated inferior results in the numerical simulation at a 60% filling time. The numerical simulation and experimental results of the micro-sized part were found to have similar appearances at a 90% filling time. The numerical simulation and experimental results of the micro-sized part were similar in appearance at a 100% filling time (Figure 6a). The difference between the experiment and numerical simulation is shown at a 60% filling time. Regardless of whether the filling stage was leading or lagging, the filling of the micro-sized blade could be determined from the situation of filling the milling blade. For the part’s blade, the numerical simulation was similar to the experimental results, but in the blade-filling situation, it numerical simulation indicated that the upper edge of the blade-filling flow was ahead of the lower edge, and the experimental blade-filling situation consisted of the blade-filling flow. The results of the numerical simulation were opposite to the experimental results. Figure 6b depicts a short shot of the filling stage of the micro-sized part with µIM using ABS between the numerical simulation and experimental results. The numerical simulation and experiment results of the micro-sized part were similar in appearance at a 30% filling time. Results indicated that for the numerical simulation of the filling stage of the micro-sized part, the appearance was inferior to the experimental results at a 60% filling time. The appearances of the numerical simulation and experimental results of the micro-sized part were similar at a 90% filling time. Results showed that the numerical simulation and experimental results of the micro-sized part were similar in appearance at a 100% filling time. The difference between the experimental results and numerical simulation results is shown at a 60% filling time (Figure 6b). Regardless of whether the filling stage was leading or lagging, it was found, based on the filling situation of the micro-sized part’s blade, that the plastic material flowed into the thin region after the micro-sized part body was filled first. For the blade part, the numerical simulation results were similar to the experimental results, but in the blade-filling situation, numerical simulation indicated that the upper edge of the blade-filling flow was ahead of the lower edge, and the experimental blade-filling situation consisted of the blade-filling flow. The numerical simulation was opposite to the experimental results. Figure 6c is a short shot of the filling stage of the numerical simulation and experimental results of the micro-sized part using PP. The simulated filling results slightly lagged behind the experimental results of the micro-sized part at a 30% filling time. The numerical simulation and experimental results of the micro-sized part were similar in appearance, with filling times of 60%, 90%, and 100%. The difference between the experimental results and numerical simulation is shown at a 60% filling time (Figure 6c). Regardless of whether the filling stage was leading or lagging, the filling of the micro-sized part’s blade could be determined from the filling situation of the milling blade. For the blade part, the numerical simulation results were similar to the experimental results, but in the blade-filling part, numerical simulation indicated that the upper edge of the blade-filling flow was ahead of the lower edge, and the experimental blade-filling part consisted of the blade-filling flow. The numerical simulation results were opposite to the experimental results. Figure 6d is a short shot of the filling stage of the numerical simulation and experimental results of the micro-sized part using PA. The filling phenomenon of the numerical simulation was slightly ahead of the experiment’s micro-sized part shape at a 30% filling time. The numerical simulation and experimental results of the micro-sized part were similar in appearance at filling times of 60%, 90%, and 100%. The experimental results and numerical simulation differed at a 60% filling time. Regardless of whether the filling stage was leading or lagging, the filling of the micro-sized blade was determined from the milling fan blade-filling situation. For the blade part, the numerical simulation results were similar to the experimental results, but for the blade-filling part, numerical simulation indicated that the upper edge of the blade-filling flow was ahead of the lower edge, and the experimental blade-filling part consisted of the blade-filling flow. For the leading edge, the numerical simulation results were opposite to the experimental results, but the leading and trailing edges of blade filling were lower than with the other materials. Figure 6e is a short shot of the numerical simulation and experimental results of the micro-sized part using ABS + PC. The numerical simulation and experimental results of the micro-sized part were similar in appearance at filling times of 30%, 60%, 90%, and 100%. A difference between the experimental results and numerical simulation of the micro-sized part appeared at a 60% filling time. Regardless of whether the filling stage was leading or lagging, the filling of the micro-sized part blade was determined from the filling situation of the millimeter fan blade. The numerical simulation results of the blade part were similar to the experimental results, but in the blade-filling part, it was found numerical simulation that the upper edge of the blade-filling flow which was slightly ahead of the lower edge. The upper edge of the micro-sized part was leading, and results of numerical simulation were opposite those of the experimental results, although the upper and lower edges of blade filling were leading and trailing, respectively, less than with the other materials.

Warpage values of the micro-sized parts for the various plastics with the numerical simulation are given in Table 4. The warpage phenomena of the micro-sized part using various polymer materials with PIM were in the order of POM > PP > ABS + PC > ABS > PA. The minimum warpage value was demonstrated at the ends of the micro-sized blade for PA, and PA was the most suitable material for the micro-sized part obtained by PIM. Therefore, PA was determined to be the most suitable material, followed by ABS, ABS + PC, and PP, whereas POM was the least suitable material in this study.

**Figure 7:**
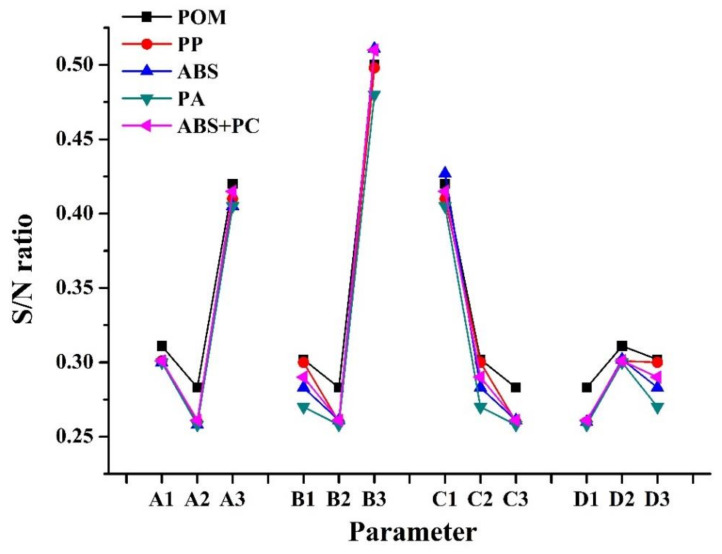
Variations in the signal-to-lose (S/N) ratio with factor level for warpage phenomenon of the micro-sized part by numerical simulation (POM/ABS/PP/PA/ABS + PC).

To minimize the warpage of the PIM-fabricated micro-sized part with various process parameters and plastics, whether in terms of experimental results or numerical simulation, the following Equation was used for the analysis to describe the smaller-the-better characteristics:0

(35) $$\frac{S}{N}=-10\log\left\{\frac{1}{n}\sum_{i=1}^{n}\frac{1}{y_{i}^{2}}\right\}$$

where *y_i_* is the measured or numerical simulation property (warpage), and n corresponds to the number of samples in each test trial. We produced a signal-to-noise (S/N) reaction diagram of warpage of the micro-sized part in PIM for the numerical simulation (Figure 7). Levels of optimal factors statistically resulting in minimum warpage of the micro-sized part using POM/ABS/PP/PA/ABS + PC materials by numerical simulation were predicted to be A2B2C3D1/A2B2C3D1/A2B2C3D1/A2B2C3D1/A2B2C3D1. These optimized factor levels of process parameters represent melt temperatures of 230/235/275/300/245 °C, mold temperatures of 85/85/50/75/60 °C, injection pressures of 50/50/25/55/40 MPa, and filling times of 1/0.5/0.1/0.1/1 s. Finally, the mold temperature was the most important process parameter, followed by the melt temperature, injection pressure, and filling time, in terms of warpage of the micro-sized part in PIM in the numerical simulation.

### 3.2. Experimentation and Measurement (of the Warpage and Mold Window)

**Figure 8:**
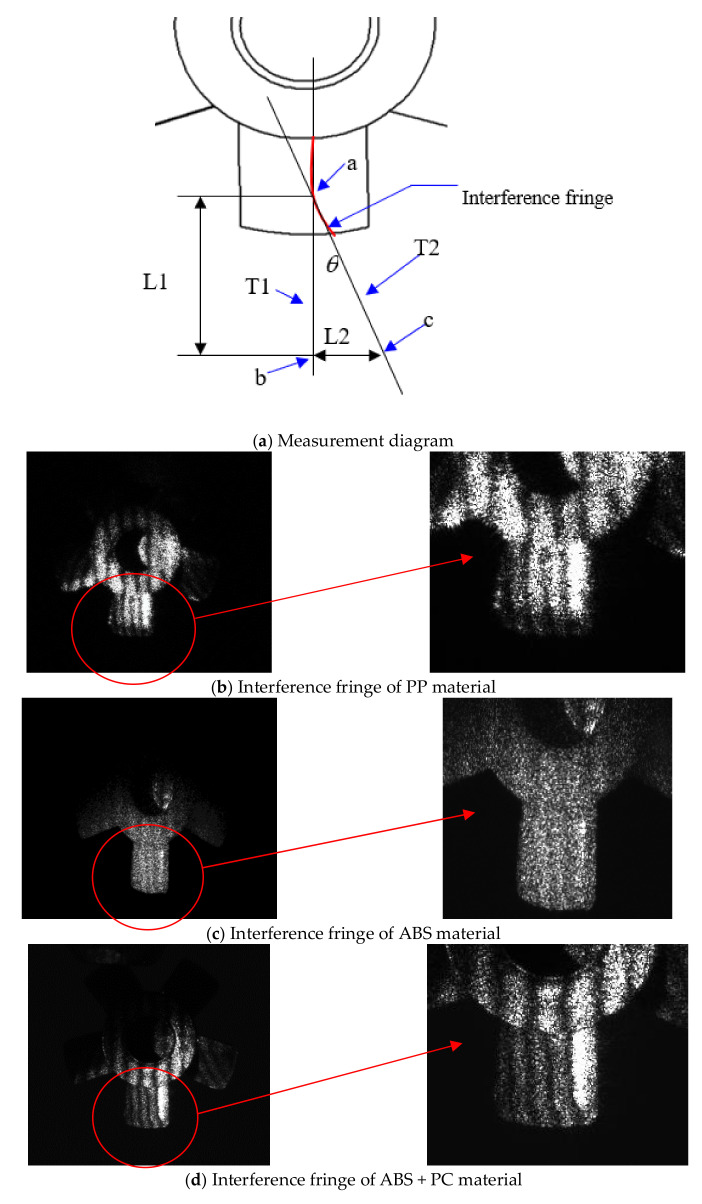

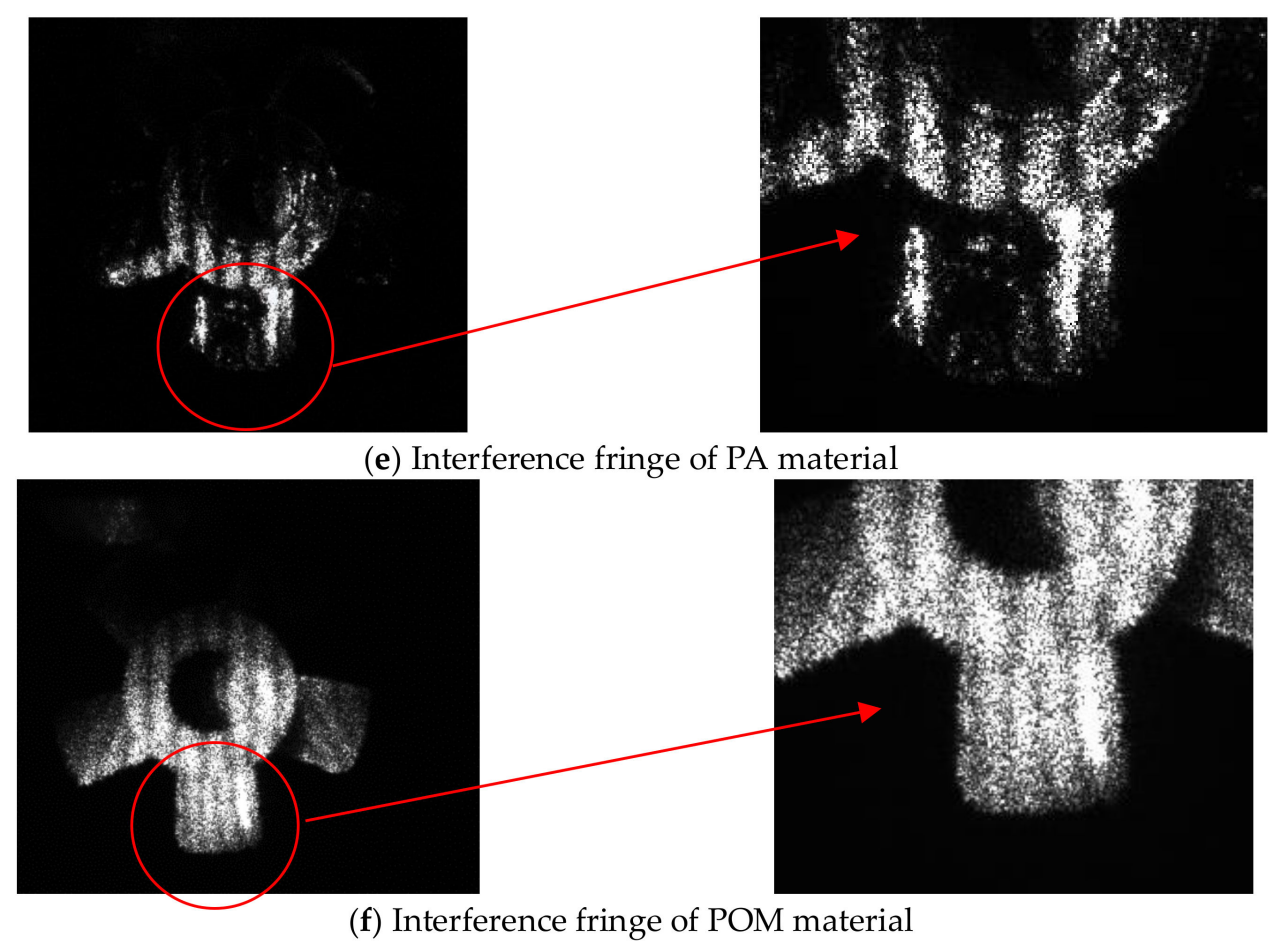
The interference fringes and situations for measurement method for micro-sized part with various plastics. (**a**) Measurement diagram; (**b**) Interference fringe of PP material; (**c**) Interference fringe of ABS material; (**d**) Interference fringe of ABS + PC material; (**e**) Interference fringe of PA material; (**f**) Interference fringe of POM material.

The measurement calculation method is shown in Figure 8a. Two tangent lines, T1 and T2, of the selected stripes were drawn to intersect at a in the Figure 8a. Take point a on T1 as b, and the distance between a and b is L1. From b, make a perpendicular to T1. The line segment intersects T2 at c, the distance from b to c is L2, and the angle between T1 and T2 is calculated using formula (34). The larger the value, the greater the amount of blade warpage.


(36) $$\theta =\tan^{-1}\left(\frac{L2}{L1}\right)$$

Figure 8b–f shows the interference fringe patterns of the PIM-fabricated micro-sized part for various plastics. Table 5 indicates the warpage value after calculation with Formula (34) from Figure 8b–f. Warpage values for various plastics after measurement and calculation are given in Table 5. The POM material exhibited the largest warpage phenomenon of the micro-sized part with PIM; followed by PP, ABS + PC, and ABS, with PA exhibiting the lowest value. Thus, PA was found to be the most suitable material, followed by ABS, ABS + PC, and PP, with POM deemed not to be a suitable material.

**Figure 9:**
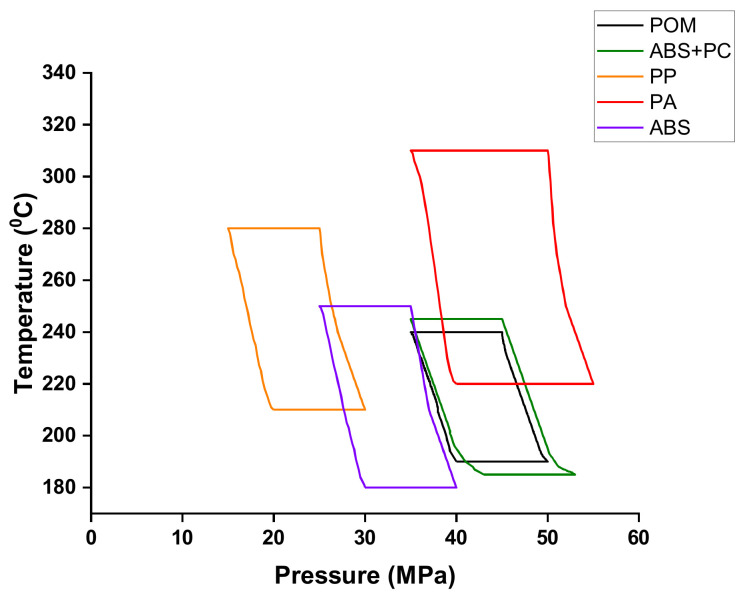
The mold window for the micro-sized part using POM, ABS, PP, PA, and ABS + PC materials by precision injection molding.

Figure 9 indicates the mold window for the PIM-fabricated micro-sized part using POM, ABS, PP, PA, and ABS + PC materials. The melt temperature exceeding the upper limit of the mold window caused material degradation. When the melt temperature reached the lower limit, IM led to a short-shot situation. When the processing value exceeded the right upper limit, processing created a flash. When the processing value was toward the lower left side, processing results were a short-shot situation. The area of the mold window had a maximum for PA, followed by ABS, ABS + PC, and PP, with POM exhibiting the minimum value. The mold results revealed that the molding process using PA was more flexible than that using ABS, ABS + PC, or PP, and POM was deemed unsuitable. This is because the melt flow index of PA (80) had the maximum value, followed by ABS (35), ABS + PC (19), and PP (12.5), with POM having the lowest value (7.4); hence, molding was easier with PA than with ABS, ABS + PC, PP, or POM.

**Figure 10:**
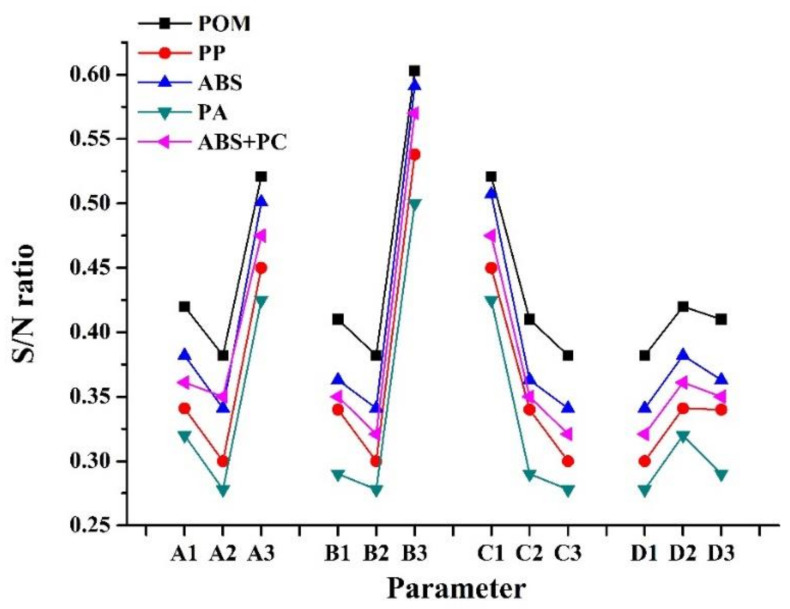
Variation of the S/N ratio with factor level for warpage phenomenon of micro-sized part by experiment (POM/ABS/PP/PA/ABS + PC).

Figure 10 indicates S/N ratios of warpage of the micro-sized part with various process parameters according to experimental results (PIM). Based on these results, optimal factor levels of process parameters that statistically resulted in minimum warpage of the micro-sized part using POM/ABS/PP/PA/ABS + PC materials for µIM were A2B2C3D1/A2B2C3D1/A2B2C3D1/A2B2C3D1/A2B2C3D1. These optimized factor levels represent melt temperatures of 230/235/275/300/245 °C, mold temperatures of 85/85/55/75/60 °C, injection pressures of 50/50/25/55/40 MPa, and filling times of 1/0.5/0.1/0.1/1 s. The results also revealed that the mold temperature was the most important process parameter affecting warpage of the PIM fabricated micro-sized part in this experiment, followed by the melt temperature, injection pressure, and filling time.

A higher mold temperature reduces the plastic’s viscosity, and this situation allows the melted plastic to more easily fill in the micro-cavities of the micro-sized part during PIM. This phenomenon caused less warpage of the micro-sized part. As the melt temperature of the plastic increases, the viscosity of the melted plastic decreases. An appropriate difference in the melt temperature and mold temperature facilitates minor differences in the temperature distribution of the plastic during the filling stage of PIM. This situation led to smaller warpage of the PIM-fabricated micro-sized part.

To sum up, results of warpage of the micro-sized part were very similar, regardless of the different materials or process parameters between the experimental results and numerical simulation with PIM.

### 3.3. Grey Theory

In this study, the analysis using grey theory was divided into two parts: optimization of predictions of experimental process parameters (grey relational analysis in grey theory) and material selection (grey decision analysis in grey theory) for PIM.

#### 3.3.1. Grey Relational Analysis in Grey-Theory-Optimal Process Parameters

For the grey relational analysis, the four axioms of grey relation and generation of a grey relation model are shown by:

(37) $$r\left(x_{0},x_{i}\right)=\frac{1}{n}\overset{n}{\underset{k=1}{\sum }}r\left(x_{0}\left(k\right),x_{i}\left(k\right)\right)$$

and

(38) $$r\left(x_{0}\left(k\right),x_{i}\left(k\right)\right)=\frac{\underset{i}{\min}\underset{k}{\max}\left|x_{0}\left(k\right)-x_{i}\left(k\right)\right|+\xi \underset{i}{\min}\underset{k}{\max}\left|x_{0}\left(k\right)-x_{1}\left(k\right)\right|}{\left|x_{0}\left(k\right)-x_{1}\left(k\right)\right|+\xi \underset{i}{\min}\underset{k}{\max}\left|x_{0}-x_{i}\left(k\right)\right|}$$

The identification coefficient, *ξ* generally has a value of 0.5.

For the experimental scheme of the grey correlation analysis, refer to Table 2 (L_9_ orthogonal table). The process parameters of PIM were (A-) melt temperature (°C), indicator 1; (B-) mold temperature (°C), indicator 2; (C-) injection pressure (MPa), indicator 3; and (D-) filling time (s), indicator 4 (Table 3). Taking PA as an example, if the mold temperature was too high, the molded micro-sized part cooled too slowly. If the mold temperature was too low, the flow rate of the melted plastic decreased after entering the mold cavity, and the rapid cooling caused short shots. This situation revealed that a mold temperature of 75 °C was best. If the melt temperature was too high, although the fluidity of the plastic was high, it was easy to cause burrs in the plastic overflow. The melt temperature being too low could cause short shots due to insufficient filling by the plastic. As the fluidity of the plastic decreased, the relative injection pressure had to be increased. Therefore, a melt temperature of 300 °C was adopted. The injection pressure was related to the injection speed and injection time. If the injection pressure was too low, the injection speed was too slow, which led to insufficient filling by the plastic, and the injection time increased. If the injection pressure was higher, the injection speed was faster, which shortened the injection time of a single micro-sized part. Therefore, an injection pressure of 50 MPa was used. The filling time was related to the injection speed and injection pressure. The greater the injection pressure, the faster the injection speed and the shorter the filling time. If one of the three factors was opposite, the other two were also opposite, so a filling time of 0.3 s was used.

Target column:

x_0_ = (300, 75, 50, 0.3)

Model indicator column:

x_1_ = (A_1_, B_1_, C_1_, D_1_) = (295, 70, 45, 0.1)

x_2_ = (A_1_, B_2_, C_2_, D_2_) = (295, 75, 50, 0.2)

x_9_ = (A_3_, B_3_, C_2_, D_1_) = (305, 80, 50, 0.1)

Using Equations ((17)–(19)), after processing the original data, we used Equation (S8) to determine the difference in the sequence size, and the results were as follows:

△_01_ = (5.00, 5.00, 10.00, 0.10)

△_02_ = (5.00, 0.00, 5.00, 0.00)

△_03_ = (5.00, 5.00, 0.00, 0.10)

△_04_ = (0.00, 5.00, 5.00, 0.10)

△_05_ = (0.00, 0.00, 0.00, 0.10)

△_06_ = (0.00, 5.00, 10.00, 0.00)

△_07_ = (5.00, 5.00, 0.00, 0.00)

△_08_= (5.00, 0.00, 10.00, 0.10)

△_09_ = (5.00, 5.00, 5.00, 0.100), maximum value = 10.00, minimum = 0.00

Taking ξ = 0.5 and using Equation (25) to calculate the grey correlation coefficient, the results were as follows:

1. *r*(x_0_(1), x_1_(1)) = 0.5000, *r*(x_0_(2), x_1_(2)) = 0.5000, *r*(x_0_(3), x_1_(3)) = 0.3333, *r*(x_0_(4), x_1_(4)) = 0.9804
2. *r*(x_0_(1), x_2_(1)) = 0.5000, *r*(x_0_(2), x_2_(2)) = 1.0000, *r*(x_0_(3), x_2_(3)) = 0.5000, *r*(x_0_(4), x_2_(4)) = 1.0000
3. *r*(x_0_(1), x_3_(1)) = 0.5000, *r*(x_0_(2), x_3_(2)) = 0.5000, *r*(x_0_(3), x_3_(3)) = 1.0000, *r*(x_0_(4), x_3_(4)) = 0.9804
4. *r*(x_0_(1), x_4_(1)) = 1.0000, *r*(x_0_(2), x_4_(2)) = 0.5000, *r*(x_0_(3), x_4_(3)) = 0.5000, *r*(x_0_(4), x_4_(4)) = 0.9804
5. *r*(x_0_(1), x_5_(1)) = 1.0000, *r*(x_0_(2), x_5_(2)) = 1.0000, *r*(x_0_(3), x_5_(3)) = 1.0000, *r*(x_0_(4), x_5_(4)) = 0.9804
6. *r*(x_0_(1), x_6_(1)) = 1.0000, *r*(x_0_(2), x_6_(2)) = 0.5000, *r*(x_0_(3), x_6_(3)) = 0.3333, *r*(x_0_(4), x_6_(4)) = 1.0000
7. *r*(x_0_(1), x_7_(1)) = 0.5000, *r*(x_0_(2), x_7_(2)) = 0.5000, *r*(x_0_(3), x_7_(3)) = 1.0000, *r*(x_0_(4), x_7_(4)) = 1.0000
8. *r*(x_0_(1), x_8_(1)) = 0.5000, *r*(x_0_(2), x_8_(2)) = 1.0000, *r*(x_0_(3), x_8_(3)) = 0.3333, *r*(x_0_(4), x_8_(4)) = 0.9804
9. *r*(x_0_(1), x_9_(1)) = 0.5000, *r*(x_0_ (2), x_9_(2)) = 0.5000, *r*(x_0_(3), x_9_(3)) = 0.5000, *r*(x_0_(4), x_9_(4)) = 0.9804

Use Equation (26) to calculate the grey correlation, β = $\frac{1}{4}$

*r*(x_0_, x_1_) = 0.5784 (that is x_1_);

*r*(x_0_, x_2_) = 0.7500 (that is x_2_);

*r*(x_0_, x_3_) = 0.7451 (that is x_3_);

*r*(x_0_, x_4_) = 0.7451 (that is x_4_);

*r*(x_0_, x_5_) = 0.9951 (that is x_5_);

*r*(x_0_, x_6_) = 0.7083 (that is x_6_);

*r*(x_0_, x_7_) = 0.7500 (that is x_7_);

*r*(x_0_, x_8_) = 0.7034 (that is x_8_);

*r*(x_0_, x_9_) = 0.6201 (that is x_9_);

Discharge the grey correlation order, arranged from largest to smallest, for PA

PA: x_5_ > x_2_ > x_7_ > x_4_ > x_3_ > x_6_ > x_8_ > x_9_ > x_1_

After the grey relational ranking, the best processing condition for PA was the fifth group (x_5_) of experimental processing parameters.

Calculation results:

POM: x_6_ > x_8_ > x_5_ > x_2_ > x_4_ > x_1_ > x_9_ > x_7_ > x_3_

ABS: x_5_ > x_7_ > x_4_ > x_2_ > x_3_ > x_6_ > x_8_ > x_9_ > x_1_

PP: x_5_ > x_2_ > x_7_ > x_4_ > x_3_ > x_6_ > x_8_ > x_9_ > x_1_

ABS + PC: x_5_ > x_2_ > x_7_ > x_4_ > x_3_ > x_6_ > x_8_ > x_9_ > x_1_

Table 6 indicates the arrangement of the grey relations of the POM/ABS/PP/PA/ABS + PC materials and the best plan. Results revealed that the best process conditions for the POM/ABS/PP/PA/ABS + PC materials were the 6/5/5/5/5 group of experimental process parameters.

Based on results of grey correlation calculations, the best process conditions in term of warpage of the micro-sized part using various plastics for grey relation calculations are shown in Table 7. Based on these results, the optimal factor levels of process parameters that statistically resulted in minimum warpage of the PIM-fabricated micro-sized parts using POM/ABS/PP/PA/ABS + PC materials were A2B3C1D1/A2B2C3D1/A2B2C3D1/A2B2C3D1/A2B2C3D1. These optimized factor levels represent melt temperatures of 230/235/275/300/245 °C, mold temperatures of 90/85/55/75/60 °C, injection pressures of 40/50/25/55/40 MPa, and filling times of 1/0.5/0.1/0.1/1 s. Therefore, the experimental result, numerical simulations, and grey relational analysis in grey theory obtained similar results for the optimal processing of the micro-sized parts for various plastics with PIM.

#### 3.3.2. Grey Decision Analysis in Grey-Theory-Suitable Material

Samples of the five material evaluation criteria are shown in Table 1, Table 8 and Table 9, and the effects on samples were processed for effect measurements. After processing, as shown in Table 8 and Table 9, the comprehensive effect was measured by Equation (31):


$$r_{11}^{\sum }:\mathrm{POM};\;r_{12}^{\sum }:\mathrm{ABS};r_{13}^{\sum }:\mathrm{PP};r_{14}^{\sum }:\mathrm{PA};r_{15}^{\sum }:\mathrm{ABS}+\mathrm{PC}$$

$$r_{11}^{\sum }=\frac{1}{7}\overset{7}{\underset{p=1}{\sum }}r_{11}^{p}=\frac{1}{7}\left(r_{11}^{1},r_{11}^{2},\ldots ,r_{11}^{7}\right)=\frac{1}{7}\left(1+1+0.71+1+0.72+1+1\right)=0.918$$

$$r_{12}^{\sum }=\frac{1}{7}\overset{7}{\underset{p=1}{\sum }}r_{12}^{p}=\frac{1}{7}\left(r_{12}^{1},r_{12}^{2},\ldots ,r_{12}^{7}\right)=\frac{1}{7}\left(0.43+0.71+1+0.85+0.63+0.38+1\right)=0.713$$

$$r_{13}^{\sum }=\frac{1}{7}\overset{7}{\underset{p=1}{\sum }}r_{13}^{p}=\frac{1}{7}\left(r_{13}^{1},r_{13}^{2},\ldots ,r_{13}^{7}\right)=\frac{1}{7}\left(0.34+1+1+1+1+1+1\right)=0.906$$

$$r_{14}^{\sum }=\frac{1}{7}\overset{7}{\underset{p=1}{\sum }}r_{14}^{p}=\frac{1}{7}\left(r_{14}^{1},r_{14}^{2},\ldots ,r_{14}^{7}\right)=\frac{1}{7}\left(0.6+0.56+0.49+0.72+0.34+1\right)=0.609$$

$$r_{15}^{\sum }=\frac{1}{7}\overset{7}{\underset{p=1}{\sum }}r_{15}^{p}=\frac{1}{7}\left(r_{15}^{1},r_{15}^{2},\ldots ,r_{15}^{7}\right)=\frac{1}{7}\left(0.43+1+1+0.99+0.91+0.5+1\right)=0.832$$

$$r_{1}=\left(r_{11}^{\sum },r_{12}^{\sum },r_{13}^{\sum },r_{14}^{\sum },r_{15}^{\sum }\right)=\left(0.918,\;0.713,\;0.906,\;0.609,\;0.832\right)$$

Consider satisfactory decision conditions,

$$r_{{ij}^{*}}^{\sum }=\min r_{{ij}^{*}}^{\sum }=\min\left\{r_{11}^{\sum },r_{12}^{\sum },\ldots ,r_{15}^{\sum }\right\}=\min\left\{0.918,0.713,0.906,0.609,0.832\right\}=r_{14}^{\sum }=0.609$$

This situation means that PA was the most satisfactory material, followed by ABS, ABS + PC, and PP, whereas POM was determined to be unsuitable. PA was the most suitable material for PIM-fabricated micro-sized parts according to simultaneous experimental results, numerical simulations, and grey theory.

### 3.4. Molded Product

**Figure 11:**
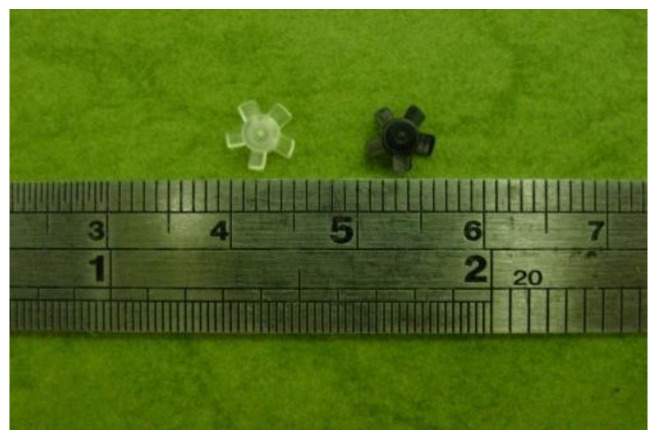
The molded micro-sized part by µIM.

Figure 11 shows the molded PIM-fabricated micro-sized parts. The goal of this study was to fabricate suitable micro-sized parts using optimal methods. The minimum warpage of the micro-sized parts was the condition for judging optimal molding. Therefore, in this study, we attempted to apply PIM to make micro-sized parts without using µIM techniques. We will employ the resulting data from this study to mass produce micro-sized parts in the future.

## 4. Conclusions

The goal of this study was to successfully fabricate micro-sized parts by PIM. We determined optimal process parameters (melt temperature, mold temperature, injection pressure, and filling time) with various materials (POM, ABS, PP, PA, and ABS + PC) for micro-sized parts using minimum warpage of the micro-sized part as judgment criterion by numerical simulations, experimental results, and grey theory.

In order to save mold-opening time, we first used numerical simulation software (Moldflow) to confirm the runner’s balance in multi-cavities of the micro-sized part flow and then simulated the warpage of the micro-sized part during PIM. According to the simulation analysis data, we employed CNC to process the mold for the PIM process. In order to obtain the best results (i.e., minimum warpage), we applied the Taguchi method and grey relational analysis in grey theory to identify the influence of each PIM process parameter.

Through numerical simulation and experimental results, the statistically optimal level of the smallest warpage of micro-sized parts using POM/ABS/PP/PA/ABS + PC materials was A2B2C3D1/A2B2C3D1/A2B2C3D1/A2B2C3D1/A2B2C3D1. These optimized process-parameter factor levels represent melt temperatures of 230/235/275/300/245 °C, mold temperatures of 85/85/50/75/60 °C, injection pressures of 50/50/25/55/40 MPa, and filling times of 1/0.5/0.1/0.1/1 s. Based on results of grey relational analysis in grey theory, the optimal factor levels of process parameters that statistically resulted in minimum warpage of the micro-sized parts using POM/ABS/PP/PA/ABS + PC materials with PIM were A2B3C1D1/A2B2C3D1/A2B2C3D1/A2B2C3D1/A2B2C3D1. These optimized factor levels represented melt temperatures of 230/235/275/300/245 °C, mold temperatures of 90/85/55/75/60 °C, injection pressures of 40/50/25/55/40 MPa, and filling times of 1/0.5/0.1/0.1/1 s. The results were very similar, regardless of whether using numerical simulations, experimental results, or grey relational analysis in grey theory for optimal processing with PIM.

To sum up, the mold temperature was the most important factor among various process parameters for PIM-fabricated micro-sized parts, whether in terms of numerical simulations, experiment results, or grey theory. Our study results suggest that micro-sized parts can be manufactured by PIM without the use of µIM, and the mold temperature required for molding does not need to be higher than the glass-transition temperature of the material. PA was the most suitable material for micro-sized parts by PIM, whether in terms of numerical simulations, experimental results (including the warpage measurement and mold window), or grey decision analysis in grey theory. The results of warpage analyses of PIM parts were very similar when using numerical simulations, experimental results, and grey theory. Ultimately, using results of this study, we could completely fabricate micro-sized parts using PIM.

## Figures and Tables

**Table 1:** Processing parameters of PIM using POM/ABS/PP/PA/ABS + PC materials.

Level Parameter	Level 1	Level 2	Level 3
A. Melt temp. (°C)	225/230/270/295/240	230/235/275/300/245	235/240/280/305/250
B. Mold temp. (°C)	80/80/50/70/55	85/85/55/75/60	90/90/60/80/65
C. Injection press. (MPa)	40/40/15/45/30	45/45/20/50/35	50/50/25/55/40
D. Filling time (s)	1/0.5/0.1/0.1/1	1.5/1/0.2/0.2/1.5	2/1.5/0.3/0.3/2

**Table 2:** The L_9_ orthogonal array used in the main experiment.

Run	Melt Temp. (A)	Mold Temp. (B)	Injection Press. (C)	Filling Time (D)
1	1	1	1	1
2	1	2	2	2
3	1	3	3	3
4	2	1	2	3
5	2	2	3	1
6	2	3	1	2
7	3	1	3	2
8	3	2	1	3
9	3	3	2	1

**Table 3:** AHP evaluation scale meaning and explanation.

Assessment Scale	Definition	Explanation
1	Equally important	The contribution of the two comparison schemes is of equal importance (equal strength)
3	Slightly important	Experience and judgment tend to prefer a certain plan (slightly stronger)
5	Quite important	Experience and judgment strongly favor a certain plan (very strong)
7	Extremely important	Very strong tendency to favor a certain plan (very strong)
9	Absolutely important	There is enough evidence to definitely favor a certain plan (very strong)
2, 4, 6, 8	Median of adjacent scales	When a compromise value is needed

**Table 4:** Numerical simulation of the warpage of micro-sized parts for PIM (POM/ABS/PP/PA/ABS + PC).

Runs.	Warpage (mm)
L1	0.427/0.412/0.450/0.410/0.430
L2	0.311/0.301/0.302/0.300/0.301
L3	0.500/0.498/0.575/0.480/0.510
L4	0.302/0.300/0.283/0.270/0.290
L5	0.283/0.260/0.261/0.258/0.261
L6	0.600/0.510/0.511/0.505/0.510
L7	0.425/0.410/0.427/0.408/0.415
L8	0.420/0.410/0.427/0.405/0.415
L9	0.560/0.500/0.552/0.498/0.523
Average	0.429/0.400/0.420/0.392/0.406

**Table 5:** Warpage of the micro-sized part in the experiment for PIM (POM/ABS/PP/PA/ABS + PC).

Runs	Warpage (mm)
L1	0.527/0.452/0.530/0.430/0.490
L2	0.420/0.341/0.382/0.320/0.361
L3	0.603/0.538/0.655/0.500/0.570
L4	0.410/0.340/0.363/0.290/0.350
L5	0.382/0.300/0.341/0.278/0.321
L6	0.710/0.550/0.591/0.525/0.570
L7	0.530/0.450/0.501/0.428/0.475
L8	0.521/0.450/0.507/0.425/0.475
L9	0.670/0.540/0.632/0.518/0.583
Average	0.505/0.440/0.500/0.413/0.466

**Table 6:** Arrangement of the grey relation of POM/ABS/PP/PA/ABS + PC materials and the best plan.

Run	Grey Correlation	Sort
1	0.7273/0.5833/0.5784/0.5784/0.5606	6/9/9/9/9
2	0.7500/0.7273/0.7500/0.7500/0.7500	4/4/2/2/2
3	0.5606/0.7273/0.7451/0.7451/0.7273	9/5/5/5/5
4	0.7273/0.7273/0.7451/0.7451/0.7273	5/3/4/4/4
5	0.8106/0.9773/0.9951/0.9951/0.9773	3/1/1/1/1
6	0.8750/0.7083/0.7083/0.7083/0.7083	1/6/6/6/6
7	0.5833/0.7500/0.7500/0.7500/0.7500	8/2/3/3/3
8	0.8523/0.6856/0.7034/0.7034/0.6856	2/7/7/7/7
9	0.6023/0.6023/0.6201/0.6201/0.6023	7/8/8/8/8

**Table 7:** The processing conditions of warpage of the micro-sized part with various plastics based on grey relational analysis.

Material	Run	Optimal Set of Processing Parameters			
		A. Melt Temp. (°C)	B. Mold Temp. (°C)	C. Injection Press. (MPa)	D. Filling Time (s)
POM	6	230	80	15	1.5
ABS	5	235	85	50	1
PP	5	235	55	25	0.1
PA	5	300	75	55	0.5
ABS + PC	5	245	60	45	1

**Table 8:** Sample of material evaluation criteria.

Target	Sample	Target Polarity	POM Scheme A	ABS Scheme B	PP Scheme C	PA Scheme D	ABS + PC Scheme E
1	Melt temp.	Minimum	195	230	230	295	240
2	Mold temp.	Minimum	70	80	50	70	55
3	Injection Press.	Minimum	15	40	15	45	30
4	Filling time	Minimum	1	1	1	1	1
5	Specific weight	Minimum	3	7	9	5	7
6	Shrinkage rate	Minimum	5	7	5	9	5
7	Tensile strength	Minimum	7	5	5	9	5

**Table 9:** Effect measurement processing of effect samples.

Target	Sample	Target Polarity	POM Scheme A	ABS Scheme B	PP Scheme C	PA Scheme D	ABS + PC Scheme E
1	Melt temp.	Minimum	1	0.85	1	0.49	0.96
2	Mold temp.	Minimum	0.71	0.63	1	0.71	0.91
3	Injection Press.	Minimum	1	0.38	1	0.34	0.5
4	Filling time	Minimum	1	1	1	1	1
5	Specific weight	Minimum	1	0.43	0.34	0.6	0.43
6	Shrinkage rate	Minimum	1	0.71	1	0.56	1
7	Tensile strength	Minimum	0.71	1	1	0.56	1

## Data Availability

The data used to support the findings of this study are included within the article.
